# Lenvatinib in combination with transarterial chemoembolization for treatment of unresectable hepatocellular carcinoma (uHCC): a retrospective controlled study

**DOI:** 10.1007/s12072-021-10184-9

**Published:** 2021-04-20

**Authors:** Zhigang Fu, Xiaowei Li, Jiaming Zhong, Xiaoxia Chen, Kunkun Cao, Ning Ding, Li Liu, Xiaoli Zhang, Jian Zhai, Zengqiang Qu

**Affiliations:** Department II of Interventional Radiology, Eastern Hepatobiliary Surgery Hospital, Shanghai, China

**Keywords:** Lenvatinib, Transarterial chemoembolization, Unresectable hepatocellular carcinoma (uHCC), Combination therapy, Monotherapy, Overall survival, Progression-free survival, Tumor response, Liver function, Adverse events

## Abstract

**Purpose:**

To compare the efficacy and safety of combined treatment with lenvatinib and transarterial chemoembolization (TACE) versus TACE only in patients with unresectable hepatocellular carcinoma (uHCC).

**Methods:**

Of the 120 patients enrolled in this study, 60 patients received treatment with TACE only, and 60 patients received TACE plus lenvatinib. We retrospectively compared the clinical outcomes including overall survival (OS), progression-free survival (PFS), and tumor response between the two groups. Both PFS and tumor response were based on the modified Response Evaluation Criteria in Solid Tumors (mRECIST). Adverse events were analyzed to assess the safety profiles.

**Results:**

The 1-year and 2-year OS rates were significantly higher in the TACE + lenvatinib group (88.4% and 79.8%) than that in the TACE group (79.2% and 49.2%, *p* = 0.047). A similar PFS benefit was observed in the TACE + lenvatinib group (1-y PFS rate: 78.4% vs. 64.7%, 2-y PFS rate: 45.5% vs. 38.0%, *p* < 0.001). The best overall objective response rate (ORR) was also better with TACE + lenvatinib treatment (ORR: 68.3% vs. 31.7%, *p* < 0.001) and disease control rate (DCR) numerically increased in the TACE + lenvatinib treatment (93.3% vs. 86.7%, *p* = 0.224). Patients’ liver function remained comparable to baseline in the TACE + lenvatinib group. The most common adverse events were decreased albumin (55.0%), hypertension (48.3%) and decreased platelet count (46.7%) in the TACE + lenvatinib group.

**Conclusions:**

Combination treatment with TACE and lenvatinib may significantly improve clinical outcomes over TACE monotherapy with a manageable safety profile for unresectable HCC. The efficacy of the combination treatment should be validated in prospective studies with a large sample size.

**Graphical abstract:**

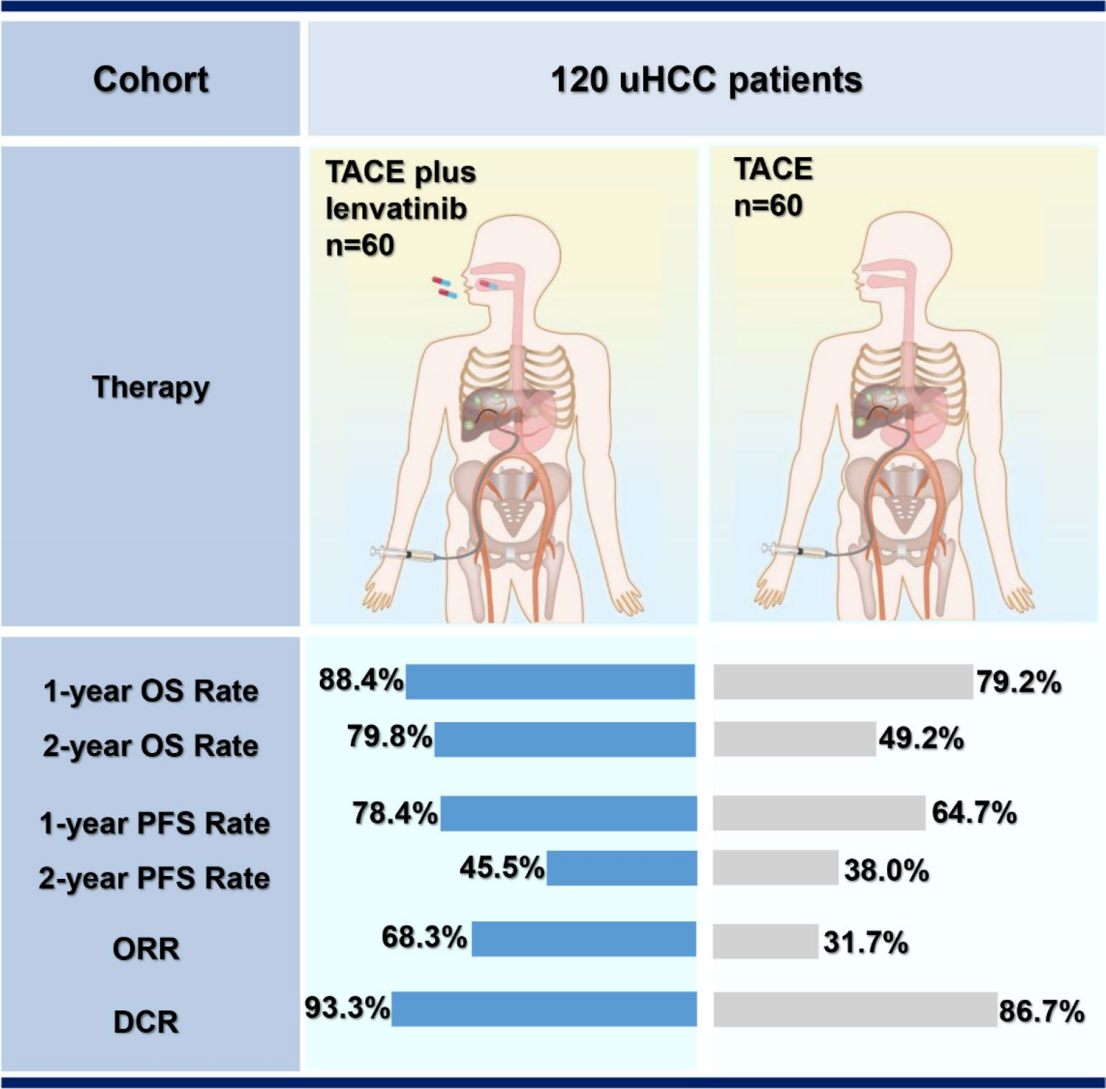

**Supplementary Information:**

The online version contains supplementary material available at 10.1007/s12072-021-10184-9.

## Introduction

Hepatocellular carcinoma (HCC) is the most common primary liver cancer and the third leading cause of cancer-associated deaths worldwide [1]. Though surgical resection is a potentially curative treatment for patients with HCC, as many as 50–70% of HCC patients are unable to undergo radical resection due to liver dysfunction, advanced tumor stage, or poor performance status, and thus, have an unfavorable prognosis [2–4].

Transarterial chemoembolization (TACE) is recommended as the standard treatment for Barcelona Clinic Liver Cancer (BCLC) B HCC [5]. In some countries, such as China, Japan and Korea, TACE is also one of the recommendations for unresectable cases [6–10]. Through targeted arterial embolization and drug administration, TACE induces ischemia and necrosis of the tumor [11, 12]. However, it also increases tumor hypoxia and activates hypoxic response signaling, thereby inducing upregulation of vascular endothelial growth factor (VEGF) and fibroblast growth factor (FGF), which can lead to tumor revascularization and progression [13, 14].

The Post-TACE and TACE-2 trials explored the combination of TACE with sorafenib, an antiangiogenic treatment, and showed negative results [15, 16]. However, a recent randomized trial, the TACTICS trial, confirmed that combination treatment with TACE and sorafenib could provide survival benefits over TACE monotherapy in patients with unresectable HCC [17]. In addition, several real-world studies have provided more clinical evidence that TACE combined with sorafenib could offer an advantage over TACE for unresectable HCC [18–21].

Currently, lenvatinib, a novel oral multi-kinase inhibitor is gaining increasing attention. By targeting multiple kinase receptors, including VEGF, FGF, and platelet-derived growth factor (PDGF) receptors, lenvatinib exerts both antiangiogenic and direct antitumor effects [22]. The latest phase 3 randomized, open-label study comparing the efficacy and safety of lenvatinib versus sorafenib, the REFLECT study, reported that the median overall survival (OS) with lenvatinib was non-inferior to that with sorafenib, but found that the progression-free survival (PFS), objective response rate (ORR) and time to progression (TTP) were significantly improved with lenvatinib over sorafenib [23]. Therefore, lenvatinib has been approved as an alternative first-line treatment for advanced HCC.

The combination of lenvatinib and TACE may have an enhanced therapeutic benefit, but to date, no data have been published regarding outcomes achieved with this combination therapy. Thus, we conducted this retrospective study to assess the efficacy and safety of combination therapy with TACE plus lenvatinib versus TACE monotherapy in patients with unresectable HCC.

## Patients and methods

### Study design and patients

Adult patients diagnosed with unresectable HCC from July 2017 to October 2019 in our hospital were retrospectively reviewed. HCC was confirmed by biopsy, cytology, dynamic computed tomography (CT) or magnetic resonance imaging (MRI) examination based on the American Association for the Study of Liver Diseases guidelines.

Patients were enrolled according to the following inclusion criteria: (a) diagnosis with unresectable HCC; (b) measurable lesions on CT or MRI; (c) liver function scored as Child–Pugh A or B; and (d) prior resection or ablation was allowed. Patients were excluded from the study if they had any of the following: (a) metastatic malignant tumors from other organs; (b) liver function scored as Child–Pugh C; (c) any contraindication for therapy with TACE or lenvatinib; and (d) treatment with other methods (including radiofrequency ablation, immune checkpoint inhibitor, iodine 125 seed implantation, etc.) simultaneously during this study. The study protocol was approved by the ethical committee of the Eastern Hepatobiliary Surgery Hospital, Second Military Medical University.

### TACE therapy

Briefly, tumor-feeding arteries were first identified by angiography. Then chemotherapeutic agents and iodized oil were injected into the arteries. The treatment regimen consisted of pirarubicin with lipiodol. All the procedures were handled by the same physician. Post-TACE evaluation and follow-up were conducted every 6–8 weeks. On- demand TACE was conducted repeatedly according to investigators’ assessment when the lesion was not fully necrotic, and the active area was greater than 50% of the baseline. Additionally, the Child–Pugh status had to remain at class A or B without evidence of hepatic decompensation (e.g., uncontrolled ascites or hepatic encephalopathy).

### Lenvatinib therapy and combination therapy

Physicians recommended the TACE plus lenvatinib treatment strategy and fully informed patients of the drug efficacy, potential adverse effects and costs. If the patient agreed to the physician’s recommendation, lenvatinib was administered 3 days later after the first TACE treatment. Patients who refused lenvatinib underwent TACE only. The dosage of lenvatinib was 12 mg (≥ 60 kg) or 8 mg (< 60 kg) once daily based on body weight. Lenvatinib was discontinued for 3 days before and then restored after each TACE session if there were no obvious symptoms caused by TACE, such as fever, nausea, vomit. While the obvious symptoms caused by TACE continued, patients would not receive lenvatinib until the symptoms released.

Dose interruptions for lenvatinib followed by reductions for lenvatinib-related toxicities (to 8 mg and 4 mg/day, or 4 mg every other day) were permitted according to the label.

### Anti-viral therapy

All patients who had hepatitis B virus (HBV) infection received antiviral medication therapy (Tenofovir or Entecavir) before the treatment and continued it as long-term treatment. The virus load was monitored during the follow-up. Patients who had hepatitis C virus (HCV) infection received Sofosbuvir treatment from the baseline.

### Safety assessment

Treatment-emergent adverse events (AEs) were assessed mainly based on the frequency and severity grade according to the criteria of the Common Terminology Criteria for Adverse Events (CTCAE, version 5.0).

AEs were recorded during follow-up of all patients, which was conducted at an interval of 6–8 weeks. Transient AEs just after TACE, such as fever, abdominal pain, and elevated liver enzymes (including aspartate transaminase (AST)/ alanine aminotransferase (ALT)), were not recorded.

### Follow-up and assessment

Out-patient follow-up was required every 6–8 weeks, and the censoring date was 2020.1.1. During each follow-up, blood tests including blood cell count, liver function tests, and levels of tumor markers (alpha-fetoprotein (AFP) and Des-gamma-carboxy prothrombin (DCP)) were performed. In addition, liver enhanced CT or MRI was performed. For both groups, tumor response was defined as the best response across all time points. PFS was defined as the time from first treatment to the progression of tumor or death caused by any reason, which was based on the Modified Response Evaluation Criteria in Solid Tumors for HCC (mRECIST). OS was defined as the time from first treatment to the death by any reason.

For evaluation of tumor markers, AFP response was evaluated in patients with the pretreatment AFP level of > 20 ng/mL. We defined early AFP response as a ≥ 20% decline in serum AFP levels at the first follow-up relative to pretreatment levels. DCP response was evaluated in patients with the pretreatment DCP level of > 40 mAU/mL. We defined early DCP response as a ≥ 20% decline in serum DCP levels at the first follow-up relative to pretreatment levels.

### Statistical analysis

Continuous data are presented as median and interquartile. The correlations between treatment category and baseline characteristics were compared using Student’s t-test for continuous variables and Fisher’s exact or *χ*^2^ test for categorical variables. Survival curves were estimated using the Kaplan–Meier method followed by log-rank test to analyze differences. Univariate and multivariate analyses based on the Cox regression model were performed to identify independent prognostic factors associated with OS. All statistical analyses were performed using SPSS, version 22.0 software, with *p* < 0.05 defining statistical significance.

## Results

### Patient characteristics

**Fig. 1:**
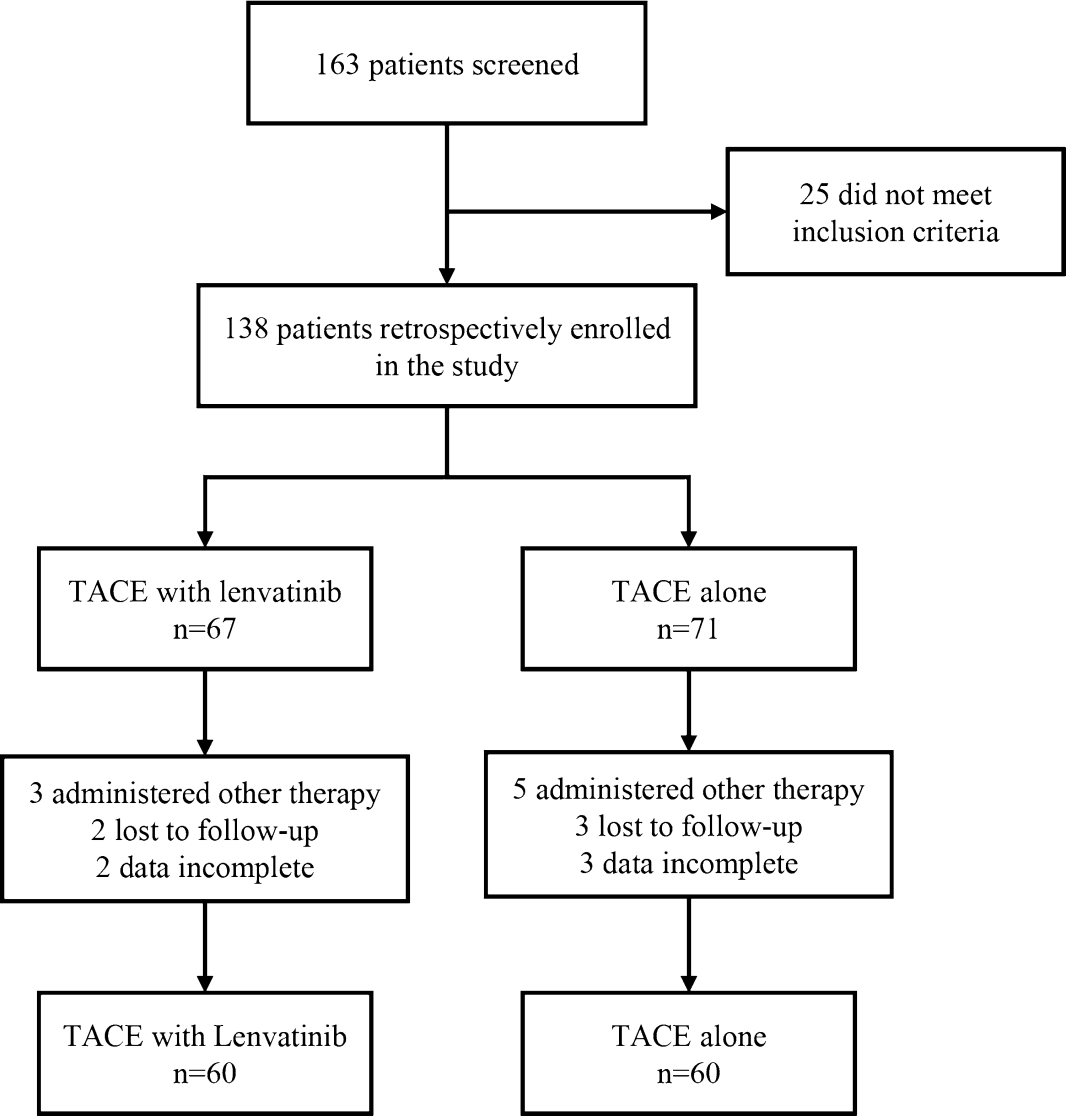
Flow diagram of patient enrollment

A flow diagram of patient enrollment is shown in Fig. 1. A total of 163 patients with unresectable HCC treated between July 2017 and October 2019 were retrospectively reviewed, and of these, 25 patients did not meet the inclusion criteria. Of the remaining 138 patients, 67 patients received combination therapy with TACE and lenvatinib and 71 received TACE only. During the follow-up, 18 patients were excluded due to administration of other therapies, loss to follow-up, or incomplete data. Finally, 120 patients were included in the current study, with 60 receiving combination therapy (TACE + lenvatinib group) and the other 60 receiving TACE monotherapy (TACE group).

The baseline clinicopathological characteristics of the enrolled patients in each group are presented in Table 1. The two groups were well balanced for almost all characteristics, including gender, Child–Pugh Score, etiology of HCC, BCLC stage, tumor number, tumor size, AFP level, total bilirubin (TB), albumin (ALB), ALBI, prothrombin time (PT), glucose, platelet (PLT) count, extrahepatic spread, and portal vein tumor thrombus. Patient age in the TACE + lenvatinib group was slightly younger than that in the TACE only group, which may be because younger patients are more willing to try novel treatments to prolong their life.

**Table 1:** Baseline demographic and clinical characteristics of patients with unresectable HCC

Characteristics	TACE + lenvatinib (*n* = 60)	TACE (*n* = 60)	*p* value
Age, median (range), years	60 (25–76)	60 (33–81)	0.011
< 65	54 (90)	43 (71.7)	
≥ 65	6 (10)	17 (28.3)	
Gender			0.168
Female	10 (16.7)	5 (8.3)	
Male	50 (83.3)	55 (91.7)	
Child–Pugh			1.000
A	56 (93.3)	57 (95.0)	
B	4 (6.7)	3 (5.0)	
Etiology			1.000
Hepatitis B	48 (80.0)	48 (80.0)	
Hepatitis C	2 (3.3)	2 (3.3)	
Non-B, Non-C	10 (16.7)	10 (16.7)	
BCLC stage			0.433
A	2 (3.3)	3 (5.0)	
B	33 (55.0)	26 (43.3)	
C	25 (41.7)	31 (51.7)	
Tumor number			0.803
Single	9 (15.0)	10 (16.7)	
Multiple	51 (85.0)	50 (83.3)	
Tumor size (mm)			0.408
< 30	9 (15.0)	6 (10.0)	
≥ 30	51 (85.0)	54 (90.0)	
AFP (ng/mL), median (Q1, Q3)	173 (7.425, 2796.75)	219.5 (11, 2169.75)	0.921
< 400	33 (55.0)	33 (55.0)	
≥ 400	27 (45.0)	27 (45)	
DCP (mAU/mL), median (Q1, Q3)	1930.5 (142, 14,030.25)	4006 (444.75, 34,988)	0.035
< 2050	31 (51.7)	22 (36.7)	
≥ 2050	29 (48.3)	38 (63.3)	
Extrahepatic spread			1.000
Yes	9 (15.0)	9 (15.0)	
No	51 (85.0)	51 (85.0)	
Portal vein tumor thrombus			0.264
Presence	21 (35.0)	27 (45.0)	
Absence	39 (65.0)	33 (55.0)	
TB (μmol/L), median (Q1, Q3)	15 (10.75, 19)	15 (11, 22)	0.411
ALB (g/L), median (Q1, Q3)	41 (38, 43)	39 (36, 43)	0.060
ALBI, median (Q1, Q3)	− 2.49 (− 2.78, − 2.23)	− 2.25 (− 2.63, − 2.05)	0.720
ALBI grade			
1	19	16	0.720
2	38	42	
3	3	2	
PT (sec), median (Q1, Q3)	11.7 (11.2, 12.45)	12.1 (11.475, 12.8)	0.094
Creatinine (μmol/L), median (Q1, Q3)	73 (64.5, 85)	70 (64, 77)	0.100
Glucose (mmol/L), median (Q1, Q3)	4.885 (4.4825, 6.695)	5.055 (4.52, 6.3625)	0.850
PLT (× 10^9^), median (Q1, Q3)	147 (113.5, 205)	144.5 (111.5, 202.75)	0.944
HAP score			
A	21 (35.0)	14 (23.3)	0.539
B	18 (30.0)	21 (35.0)	
C	13 (21.7)	17 (28.3)	
D	8 (13.3)	8 (13.3)	

Data are presented as *n* (%) or median (Q1, Q3). Q1 and Q3 are 25th percent and 75th percent of interquartile range. There were nine patients with a single lesion in the combination group. Three of them had portal vein tumor thrombus (PVTT) only; three of them had extrahepatic spread (EHS) only; and one had both PVTT and EHS. That is why only two patients were staged as BCLC A

*TACE* transcatheter arterial chemoembolization, *AFP* alpha-fetoprotein concentration, *DCP* Des-gamma-carboxy prothrombin, *TB* total bilirubin, *ALB* albumin, *ALBI grade* albumin-bilirubin grade, *PT* prothrombin time, *PLT* platelet, *HAP score* hepatoma arterial-embolization prognostic score

### Efficacy outcomes

Fifty-three patients (88.3%) started lenvatinib within 3 days after the first TACE, while six patients (10.0%) did on the day 4–7. Only one patient started lenvatinib on the day 14 due to abdominal pain and fever.

The median follow-up durations for the TACE + lenvatinib and TACE groups were 11.6 and 17.5 months, respectively. The censoring date was 2020.1.1. The median lenvatinib treatment duration was 8.23 (4, 11.8) months.

**Fig. 2:**
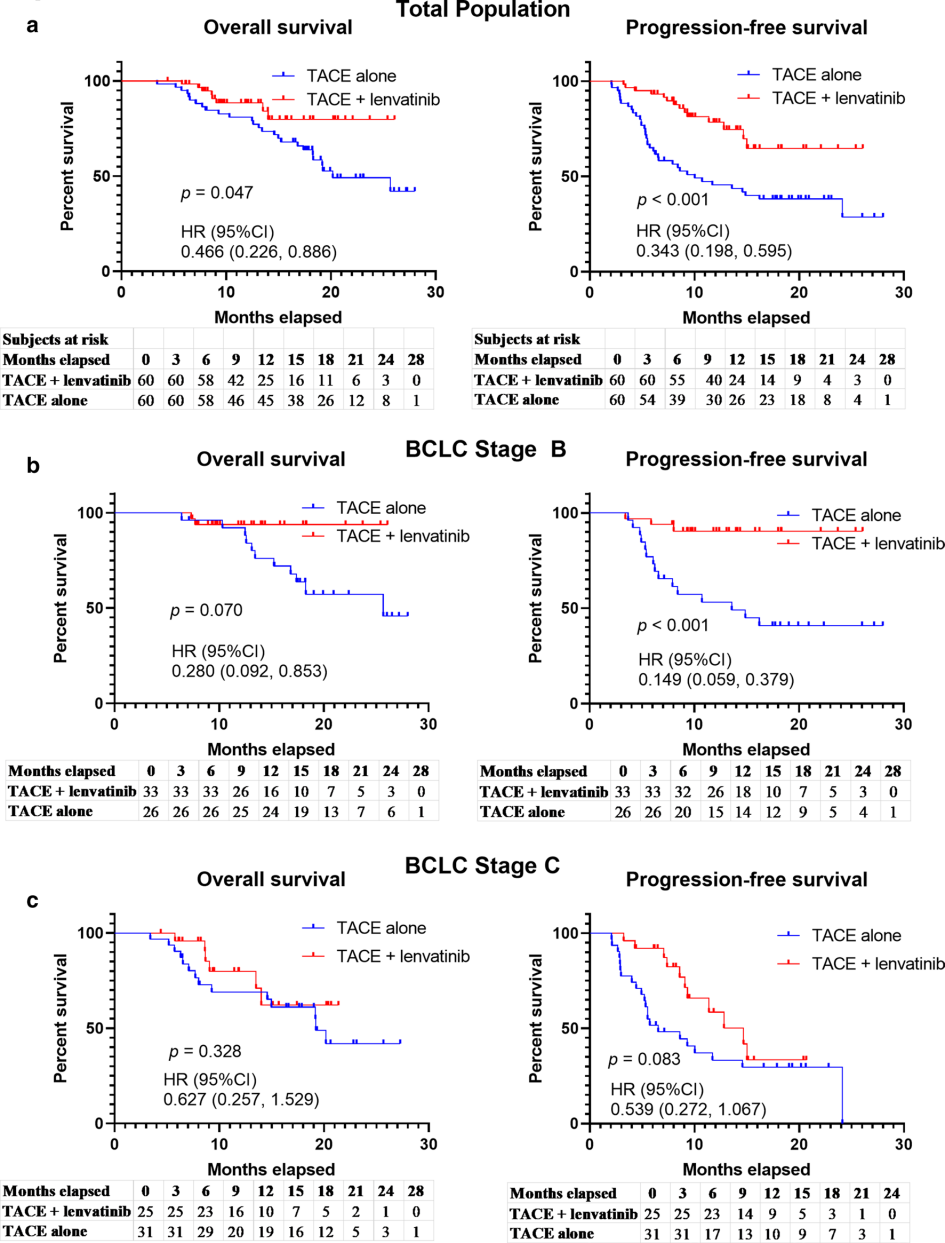
Overall and progression-free survival with different therapies. **a** The OS and PFS in the total population. **b** The OS and PFS in BCLC stage B patients. **c** The OS and PFS in BCLC stage C patients

The 1-y and 2-y OS rates were 88.4% (95% confidence interval [CI] 77.9–94.3%) and 79.8% (95% CI 68.0–88.0%), respectively, for the TACE + lenvatinib group and 79.2% (95% CI 67.3–87.6%) and 49.2% (95% CI 37.0–61.5%), respectively, for the TACE group. Thus, OS was significantly prolonged with the combination treatment (*p* = 0.047, hazard ratio [HR] = 0.466, 95% CI 0.226–0.886; Fig. 2a). PFS was also longer for patients in the TACE + lenvatinib group than for those in the TACE group (1-y PFS rate: 78.4% (95% CI 66.5–86.9%) vs. 64.7% (95% CI 52.1–75.9%), 2-y PFS rate 45.5% (95% CI 33.6–58.0%) vs. 38.0% (95% CI 26.8–50.7%), *p* < 0.001, HR = 0.343, 95% CI 0.198–0.595; Fig. 2a).

The best tumor response rates are shown in Table 2. According to the mRECIST criteria, the ORR in the TACE + lenvatinib group was 68.3%, which was dramatically higher than the ORR of 31.7% observed in the TACE group (*p* < 0.001). In addition, 56 cases in the TACE + lenvatinib group and 52 cases in the TACE group achieved disease control (DCR 93.3% vs. 86.7%, *p* = 0.224).

**Table 2:** Best tumor response in the total and subgroups

	Total		*p* value	BCLC B		*p* value	BCLC C		*p* value
	TACE + lenvatinib (*n* = 60)	TACE (*n* = 60)		TACE + lenvatinib (*n* = 33)	TACE (*n* = 26)		TACE + lenvatinib (*n* = 25)	TACE (*n* = 31)	
CR	6 (10.0%)	3 (5.0%)		4 (12.1%)	2 (7.7%)		0 (0)	0 (0)	
PR	35 (58.3%)	16 (26.7%)		19 (57.6%)	8 (30.8%)		16 (64.0%)	7 (22.6%)	
SD	15 (25.0%)	33 (55.0%)		9 (27.3%)	15 (57.7%)		6 (24.0%)	17 (54.8%)	
PD	4 (6.7%)	8 (13.3%)		1 (3.0%)	1 (3.8%)		3 (12.0%)	7 (22.6%)	
ORR	41 (68.3%)	19 (31.7%)	< 0.001	23 (69.7%)	10 (38.5%)	0.016	16 (64.0%)	7 (22.6%)	0.002
DCR	56 (93.3%)	52 (86.7%)	0.224	32 (97.0%)	25 (96.2%)	1.000	22 (88.0%)	24 (77.4%)	0.499

Data are presented as *n* (%)

*TACE* transcatheter arterial chemoembolization, *CR* complete response, *PR* partial response, *SD* stable disease, *PD* progressive disease, *ORR* objective response rate, *DCR* disease control rate

Within the TACE + lenvatinib group, subgroup analysis for both BCLC stage B and stage C indicated the benefit trend was generally consistent with the total population (Fig. 2b, c; Table 2).

### Change of liver function

The Child–Pugh score was used to evaluate the hepatic functional reserve in both groups between baseline and the first follow-up after treatment. The baseline situation of Child–Pugh grade A was well balanced between the two groups. There was no significant change between baseline and the first follow-up after treatment in either group (TACE + lenvatinib group: *p* = 0.697; TACE group: *p* = 0.309, Table 3). Liver function deterioration to grade B was observed for three patients in the TACE + lenvatinib group and one patient in the TACE group. Most patients maintained their liver function at the first follow-up.

**Table 3:** Change of liver function

	TACE + lenvatinib (*n* = 60)		*p* value	TACE (*n* = 60)		*p* value
	Baseline	First follow-up after treatment		Baseline	First follow-up after treatment	
Child–Pugh grade						
A B	56 4	57 3	0.697	57 3	59 1	0.309

*TACE* transcatheter arterial chemoembolization

### Change of tumor marker expression

**Fig. 3:**
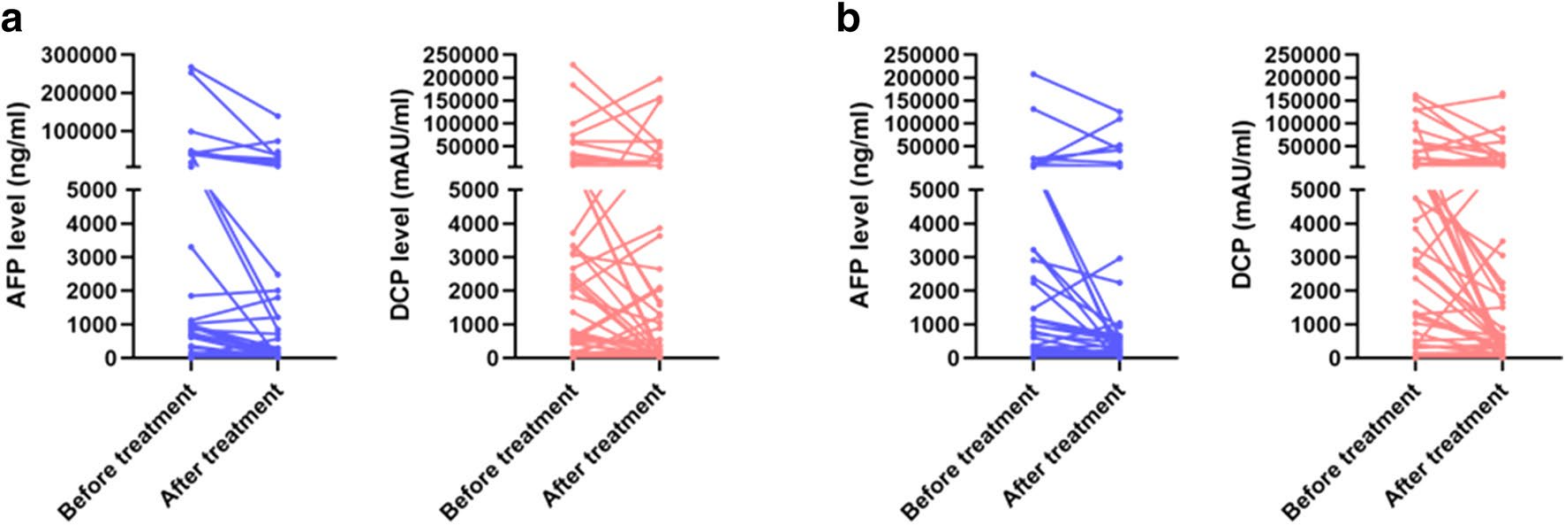
Changes in AFP and DCP levels from baseline to 6–8 weeks after the first TACE treatment. **a** Changes in AFP and DCP levels in the TACE + lenvatinib group. **b** Changes in AFP and DCP levels in the TACE group. The AFP and DCP levels decreased significantly in most patients of both groups

The changes in tumor marker levels from baseline to the first follow-up after treatment were evaluated in both groups. The AFP and DCP levels were significantly decreased after treatment in most patients in both groups (Fig. 3). As shown in Table 4, in the TACE + lenvatinib group, the median baseline AFP was 173.0 ng/mL, while this level decreased dramatically to 31.5 ng/mL at the first follow-up (*p* < 0.001). In the TACE group, the median baseline AFP was 219.5 ng/mL, while this level decreased dramatically to 53.0 ng/mL at the first follow-up (*p* = 0.023). For DCP, the median baseline level was 1930.5 mAU/mL, and this level decreased dramatically to 347 mAU/mL at the first follow-up (*p* = 0.027) in the TACE + lenvatinib group. In the TACE group, the median baseline DCP level was 4006.0 mAU/mL, and this level decreased dramatically to 638.0 mAU/mL at the first follow-up (*p* = 0.001).

**Table 4:** Change of tumor marker expression

	TACE + lenvatinib (*n* = 60)		*p* value	TACE (*n* = 60)		*p* value
	Baseline	First follow-up after treatment		Baseline	First follow-up after treatment	
AFP (ng/mL), median (Q1, Q3)	173.0 (7.4, 2796.8)	31.5 (4.6, 1210.0)	< 0.001	219.5 (11.0, 2169.8)	53.0 (7.0, 584.0)	0.023
DCP (mAU/mL), median (Q1, Q3)	1930.5 (142.0, 14,030.3)	347 (31.0, 5728.0)	0.027	4006.0 (444.8, 34,988.0)	638.0 (129.0, 12,253.0)	0.001

*TACE* transcatheter arterial chemoembolization, *AFP* alpha-fetoprotein concentration, *DCP* Des-gamma-carboxy prothrombin

AFP > 20 ng/mL was observed in 40 patients in the TACE + lenvatinib group and 41 patients in the TACE group at baseline, with no significant difference between the 2 groups (*p* = 0.845). At the first follow-up (6–8 weeks after the first TACE procedure), 31 patients (77.5%) achieved AFP response in the TACE + lenvatinib group, and 28 patients (68.3%) did in the TACE group (*p* = 0.352). DCP > 40 mAU/mL was observed in 49 patients and 53 patients in the TACE + lenvatinib group and TACE group, respectively, at baseline (*p* = 0.306). Similarly, a DCP response was observed in 33 patients (67.3%) in the TACE + lenvatinib group and 38 patients (71.7%) in the TACE group (*p* = 0.633).

### TACE interval time

In the TACE group, the median interval between TACE treatment was 74.7 days, and 36 patients (60.0%) received TACE more than twice (Table 5). In the TACE + lenvatinib group, only 24 patients (40.0%) received TACE more than twice, and the median interval between each TACE treatment was significantly longer at 103.3 days (*p* = 0.004). Thus, the addition of lenvatinib to TACE therapy could significantly decrease the number of TACE sessions required and extend the interval time, which could be helpful for maintaining liver function.

**Table 5:** Number of TACE procedures and treatment intervals

	TACE + lenvatinib (*n* = 60)	TACE (*n* = 60)	*p* value
Number of TACE procedures, *n* (%)			
1	0	4 (6.7%)	
2	36 (60.0%)	20 (33.3%)	
3	20 (33.3%)	22 (36.7%)	
4	4 (6.7%)	14 (23.3%)	
Median interval between TACE, days (SD) (days)	103.3 (76.3)	74.7 (27.1)	0.004

*TACE* transcatheter arterial chemoembolization

### Prognostic factors for OS in the TACE + lenvatinib group

Univariate and multivariate analyses based on the Cox regression model were performed to identify independent prognostic factors associated with OS (Suppl. Table 1). Univariate log-rank test analysis showed that OS was associated with treatment option (*p* = 0.041), tumor metastasis (*p* = 0.040), and portal vein tumor thrombus (*p* = 0.036). On the multivariate analysis, only treatment option was identified as an independent prognostic factor for OS (*p* = 0.048, HR = 2.180, 95% CI 1.017–4.917).

We further identified whether AFP or DCP response was an independent prognostic factor associated with OS. However, no significant association was found between either AFP or DCP response and OS (AFP response *p* = 0.388, DCP response *p* = 0.281, Suppl. Table 2). These results should be confirmed in large-scale randomized, controlled trials.

### Subsequent treatment

As shown in Table 6, 5 patients (35.7%) received subsequent treatments after 14 patients progressed in the TACE + lenvatinib group, while 23 patients (37 patients progressed, 62.2%) in the TACE group. In the combination group, two patients added a PD-1 inhibitor to primary treatment after progression. In the TACE group, most patients continued TACE after progression until unTACEable [17].

**Table 6:** Subsequent treatment

	TACE + lenvatinib (*n* = 14)		TACE (*n* = 37)	
Accepted subsequent treatments	5	35.7%	23	62.2%
TACE + lenvatinib + PD-1	2	14.3%	0	0.0%
Regorafenib + Nivolumab	1	7.1%	1	2.7%
Radiotherapy	1	7.1%	0	0.0%
TACE + PD-1	1	7.1%	1	2.7%
TACE	0	0.0%	17	45.9%
Radiotherapy + TACE	0	0.0%	2	5.4%
Ablation + TACE	0	0.0%	1	2.7%
FOLFOX4	0	0.0%	1	2.7%
Best Supportive Care	9	64.3%	14	37.8%

Data are presented as *n* (%)

*TACE* transcatheter arterial chemoembolization, *PD-1* programmed cell death-1 inhibitor

### Safety outcomes

Treatment-emergent AEs were assessed mainly based on frequency and severity grade according to CTCAE, version 5.0. Almost all patients suffered from transient fever, abdominal pain, and elevated liver enzymes (including AST/ALT) after TACE, which resolved within a short time for most patients. Hence, we did not summarize these transient AEs caused by the procedure.

As shown in Table 7, the most common AEs of all grade in the TACE + lenvatinib group were decreased albumin (55.0%), hypertension (48.3%) and decreased platelet count (46.7%). In addition, the most common grade 3/4 AE was hypertension (23.3%). Thus, the combination treatment had an acceptable safety profile without unexpected safety signals.

**Table 7:** Treatment emergent adverse events

Adverse events	TACE + lenvatinib (*n* = 60)		TACE (*n* = 60)	
	All grades *n* (%)	Grade 3/4 *n* (%)	All grades *n* (%)	Grade 3/4 *n* (%)
Decreased albumin	33 (55.0%)	0 (0.0%)	23 (38.3%)	0 (0.0%)
Hypertension	29 (48.3%)	14 (23.3%)	0 (0.0%)	0 (0.0%)
Decreased PLT	28 (46.7%)	8 (13.3%)	30 (50.0%)	5 (8.3%)
Elevated AST	23 (38.3%)	2 (3.3%)	20 (33.3%)	0 (0.0%)
Elevated GGT	17 (28.3%)	1 (1.7%)	22 (36.7%)	4 (6.7%)
Decreased WBC	17 (28.3%)	2 (3.3%)	13 (21.7%)	3 (5.0%)
Elevated ALT	14 (23.3%)	1 (1.7%)	11 (18.3%)	0 (0.0%)
Bleeding (gingiva)	13 (21.7%)	1 (1.7%)	0 (0.0%)	0 (0.0%)
Elevated TB	11 (18.3%)	1 (1.7%)	15 (25.0%)	0 (0.0%)
Diarrhea	11 (18.3%)	0 (0.0%)	0 (0.0%)	0 (0.0%)
Fatigue	10 (16.7%)	0 (0.0%)	0 (0.0%)	0 (0.0%)
Dysphonia	9 (15.0%)	0 (0.0%)	0 (0.0%)	0 (0.0%)
Hand-foot skin reaction	7 (11.7%)	0 (0.0%)	0 (0.0%)	0 (0.0%)
Elevated creatinine	2 (3.3%)	0 (0.0%)	0 (0.0%)	0 (0.0%)
Prolonged PT	2 (3.3%)	0 (0.0%)	5 (8.3%)	0 (0.0%)
Albuminuria/Proteinuria	2 (3.3%)	2 (3.3%)	0 (0.0%)	0 (0.0%)
Decreased appetite	2 (3.3%)	0 (0.0%)	0 (0.0%)	0 (0.0%)
Joint pain	2 (3.3%)	0 (0.0%)	0 (0.0%)	0 (0.0%)
Edema	1 (1.7%)	0 (0.0%)	0 (0.0%)	0 (0.0%)
Constipation	1 (1.7%)	0 (0.0%)	0 (0.0%)	0 (0.0%)

*TACE* transcatheter arterial chemoembolization, *PLT* platelet, *AST* aspartate transaminase, *GGT* γ-glutamyl transpeptidase, *WBC* white blood cell, *ALT* alanine aminotransferase, *TB* total bilirubin, *PT* prothrombin time

In the TACE group, the most common AEs were decreased platelet count (50.0%), elevated γ-glutamyl transpeptadase (GGT, 36.7%) and elevated AST (33.3%), while Grade 3/4 AEs were rare.

AEs that occurred more frequently in the TACE + lenvatinib group than in the TACE group included hypertension, bleeding (gingiva), diarrhea, fatigue, dysphonia, and hand-foot skin reaction, and these AEs were likely due to the effects of lenvatinib.

## Discussion

The present study retrospectively compared the efficacy and safety of TACE plus lenvatinib combination therapy with those of TACE monotherapy in patients with unresectable HCC. The results showed that TACE in combination with lenvatinib contributed to longer OS and PFS. The treatment option of combination therapy was identified as an independent predictive factor for improved prognosis. Moreover, combination therapy also showed better ORR. In addition, the median interval between each TACE procedure was significantly longer in the combination therapy group than in the TACE group. Liver function could be well maintained after combination therapy based on our early detection. The safety profile of the combination therapy was acceptable without unexpected safety signals. Together, these results indicated that combination therapy of TACE plus lenvatinib provided more clinical benefits than TACE alone in unresectable HCC patients with a manageable safety profile.

TACE is the standard treatment for intermediate stage HCC [5]. For a large portion of patients who are ineligible for hepatic resection due to an extremely large tumor size, unsuitable tumor position, a bi-lobar multifocal tumor, or rejection of surgery, TACE is the most important and widely used palliative treatment. In some countries, it is even recommended as one of the candidate options for intermediate-advanced stage [6–10]. However, the efficacy of TACE monotherapy for unresectable HCC remains limited with unsatisfactory effectiveness and duration of disease control. One reason for these limitations is the hypoxic environment created within the tumor by TACE, which leads to high angiogenic factor secretion after the procedure. Under this circumstance, TACE combined with systemic anti-angiogenic therapy may partially address this problem. Based on this scientific rationale, several studies have explored TACE and antiangiogenic agent combination. The TACTICS trial, which studied TACE plus sorafenib in unresectable HCC patients, showed a significantly better PFS and numerically longer OS with combination therapy versus TACE [17, 24]. Several real-world studies have provided more clinical evidence that such combination therapy could offer an advantage over TACE for unresectable HCC patients [18–21]. However, negative results were reported by other two trials, the Post-TACE and TACE-2 trials [15, 16]. The discrepancies may have resulted from the short treatment duration and timing of sorafenib administration in the latter two trials [25].

Multiple systemic agents have recently been approved for the first-line and second-line treatment for advanced HCC, which increases the potential opportunities for combining locoregional and systemic therapies. The REFLECT trial met the primary endpoint of noninferiority of lenvatinib versus sorafenib, a shocking result that produced the greatest breakthrough in HCC treatment in the last 10 years. Hence, lenvatinib was approved as an alternative first-line treatment for advanced HCC in the USA, EU, China, and Japan [8, 26–28]. To our knowledge, the present study is the first retrospective study to explore the efficacy and safety of TACE plus lenvatinib combination therapy for unresectable HCC. Our study showed this novel combination provided improved prognosis compared with TACE monotherapy. The combination treatment significantly prolonged OS (*p* = 0.047, HR = 0.466, 95% CI 0.226–0.886) and PFS (*p* < 0.001, HR = 0.343, 95% CI 0.198–0.595) than TACE alone. The results were consistent with an article from Llovet, et al., which showed that the threshold of PFS HR ≤ 0.6 was correlated with OS in advanced HCC [29]. These encouraging survival benefits may be induced by the following potential mechanism. First, as a novel oral inhibitor, lenvatinib simultaneously suppresses the activity of factors involved in tumor angiogenesis while also suppressing tumor proliferation signals via the VEGF and FGF receptors [22, 23]. Because of these properties, lenvatinib is an extremely effective inhibitor of angiogenesis. Most patients (88.3%) in our study successfully received lenvatinib treatment within 3 days after the first TACE to suppress the upregulation of VEGF and FGF. Second, relatively good tolerance to lenvatinib results in low rates of discontinuation and dose reduction as well as longer administration times [30]. A sufficient duration of lenvatinib treatment may explain the high response rate to combination therapy in our study. Moreover, lenvatinib may normalize tumor vessels, facilitating the distribution and delivery of anticancer drugs such as pirarubicin [31–33].

In addition, the median interval between each TACE procedure was significantly longer in the combination group than in the TACE group. As is well established, repeat TACE can impair liver function. A recent study reported that advanced HCC patients treated with lenvatinib showed maintained or improved liver functional reserves after 4 and 12 weeks [34]. Lenvatinib combined with TACE could decrease the need for repeated TACE procedures and maintain liver function, which would contribute to better clinical outcomes. In our study, liver function after treatment was also preserved well.

Certain underlying limitations need to be considered when interpreting our results. Firstly, the retrospective nature of our study results in various bias affecting survival outcomes. Patients' willing to choose the lenvatinib treatment would have a potential effect on the outcome of results. Also, the sample size was relatively small. The results should be interpreted with caution and need to be further confirmed in randomized controlled trials.

Secondly, the enrolled unresectable HCC population was heterogeneous. The treatment landscape of HCC is quite different for stage C patients between eastern and western countries [5–10]. According to Chinese consensus or guidelines [6–8], TACE is an optimal treatment for CNLC stages IIIa and an alternative treatment for CNLC IIIb HCC who are equal to BCLC stage C. However, there is no phase 3 trial of TACE in BCLC stage C, and all the evidences were retrospective. Although there are several systemic treatment choices in recent years, TACE is still widely used for BCLC stage C patients due to limited affordability to novel drugs after discussion based on guidelines, benefit trials and potential AEs in clinical practice. Subgroup analysis for both BCLC stage B and stage C indicated the benefit trend was generally consistent with the total population in our study. However, due to the small sample size and imbalanced clinical characteristics in subgroups, the results should be cautiously interpreted and should be validated in large sample RCTs. In addition, it would also be very helpful if lenvatinib alone could be added as a control treatment.

Additionally, the median OS was immature. Follow-up in our study should be continued.

We investigated the role of tumor markers AFP response or DCP response, which may not be proper prognostic factors. Other precise biomarkers should be explored for this combination treatment.

In conclusion, our study suggests combination treatment with TACE and lenvatinib offers a superior trend for prolonging the survival of patients with unresectable HCC compared with TACE monotherapy. Consistently, Kudo et al. suggested that early introduction of lenvatinib for certain cases receiving TACE might contribute to an improvement of prognosis [35, 36]. However, the combination timing, duration of administration, and sequencing of the systemic agent with TACE vary and remain controversial in the reported clinical trials. Future large sample, randomized, controlled trials are needed to confirm our results and address these issues.

## Supplementary Information

Below is the link to the electronic supplementary material.

Supplementary file1 (DOCX 21 KB)

## Data Availability

All data and materials generated and/or analyzed during the current study are available from the corresponding author upon reasonable request.
