# Supplementary material for: Lenvatinib in combination with transarterial chemoembolization for treatment of unresectable hepatocellular carcinoma (uHCC): a retrospective controlled study
Source: Hepatol Int. 2021 Apr 20;15(3):663–75. doi: 10.1007/s12072-021-10184-9 (PMC8286947; doi:10.1007/s12072-021-10184-9)
Supplement: Supplementary file 1 — Supplementary file1 (DOCX 21 KB) [file 12072_2021_10184_MOESM1_ESM.docx]

**Supplementary Table 1. Univariate and multivariate analyses of prognosis factors (baseline) for OS**

| Variables | Univariate analysis | | Multivariate analysis | |
| --- | --- | --- | --- | --- |
|  | HR (95% CI) | p | HR (95% CI) | p |
| Treatment option (combined therapy vs. TACE only) | 2.316 (1.034-5.185) | ***0.041*** | 2.180[1.017-4.917] | ***0.048*** |
| Gender (female vs. male) | 1.280 (0.495-3.310) | 0.611 |  |  |
| Age (<65 vs ≥65 years) | 0.463 (0.178-1.204) | 0.114 |  |  |
| Child-Pugh (A vs. B) | 1.962 (0.682-5.643) | 0.211 |  |  |
| BCLC stage (A&B vs. C) | 1.842 (0.984-3.447) | 0.056 |  |  |
| Tumor number (single vs. multiple) | 1.599 (0.561-4.558) | 0.379 |  |  |
| Tumor size (<30 vs. ≥30 mm) | 1.820 (0.556-5.959) | 0.322 |  |  |
| AFP (<400 vs. ≥400 ng/mL) | 1.933 (0.976-3.831) | 0.059 |  |  |
| DCP (<2050 vs. ≥2050 mAU/mL) | 1.242 (0.620-2.486) | 0.541 |  |  |
| Metastasis (absent vs. presence) | 2.176 (1.037-4.564) | ***0.040*** | 1.843 (0.844-4.028) | 0.125 |
| Portal vein tumor thrombus (absent vs. present) | 2.059 (1.049-4.040) | ***0.036*** | 1.669 (0.816-3.414) | 0.161 |

TACE: transcatheter arterial chemoembolization. AFP: alpha -fetoprotein concentration; DCP: Des-gamma-carboxy prothrombin.

**Supplementary Table 2 Univariate analysis of prognosis factors (tumor marker response) for OS**

**Supplementary Table 2A**

| Variables | Univariate Analysis | |
| --- | --- | --- |
|  | HR (95% CI) | p value |
| AFP response (No vs Yes) | 1.197 (0.796, 1.799) | 0.388 |

**Supplementary Table 2B**

| Variables | Univariate Analysis | |
| --- | --- | --- |
|  | HR (95% CI) | p value |
| DCP response (No vs Yes) | 1.221 (0.85, 1.754) | 0.281 |

AFP: alpha -fetoprotein concentration; DCP: Des-gamma-carboxy prothrombin.
